# Advances and potential of omics studies for understanding the development of food allergy

**DOI:** 10.3389/falgy.2023.1149008

**Published:** 2023-03-24

**Authors:** Sayantani B. Sindher, Andrew R. Chin, Nima Aghaeepour, Lawrence Prince, Holden Maecker, Gary M. Shaw, David K. Stevenson, Kari C. Nadeau, Michael Snyder, Purvesh Khatri, Scott D. Boyd, Virginia D. Winn, Martin S. Angst, R. Sharon Chinthrajah

**Affiliations:** ^1^ Sean N. Parker Center for Allergy and Asthma Research at Stanford University, Palo Alto, CA, United States; ^2^ Department of Anesthesiology, Perioperative and Pain Medicine, Stanford University School of Medicine, Palo Alto, CA, United States; ^3^ Department of Pediatrics, Stanford University School of Medicine, Palo Alto, CA, United States; ^4^ Department of Biomedical Data Science, Stanford University School of Medicine, Palo Alto, CA, United States; ^5^ Department of Medicine, Institute for Immunity, Transplantation and Infection, Stanford University School of Medicine, Palo Alto, CA, United States; ^6^ Department of Genetics, Stanford University, Palo Alto, CA, United States; ^7^ Department of Pathology, Stanford University, Palo Alto, CA, United States; ^8^ Department of Obstetrics and Gynecology, Stanford University School of Medicine, Palo Alto, CA, United States

**Keywords:** food allergy (FA), genomics, proteomics, transcriptomics, metabolomics, disease development

## Abstract

The prevalence of food allergy continues to rise globally, carrying with it substantial safety, economic, and emotional burdens. Although preventative strategies do exist, the heterogeneity of allergy trajectories and clinical phenotypes has made it difficult to identify patients who would benefit from these strategies. Therefore, further studies investigating the molecular mechanisms that differentiate these trajectories are needed. Large-scale omics studies have identified key insights into the molecular mechanisms for many different diseases, however the application of these technologies to uncover the drivers of food allergy development is in its infancy. Here we review the use of omics approaches in food allergy and highlight key gaps in knowledge for applying these technologies for the characterization of food allergy development.

## Introduction

The prevalence of food allergy (FA) is rising globally, with recent estimates reporting that about 8% of the population in the US has FA (1, 2). This comes with a substantial impact on quality of life and economic burden, costing the U.S. almost $25 billion per year (3, 4). FA is usually driven by the immune system mounting a T helper 2 (Th2) cell-mediated response against normally harmless food antigens, leading to the release of Th2 cytokines such as IL-4, IL-5, and IL-13, which recruit and activate immunoglobulin E (IgE) producing B cells, mast cells, and eosinophils. Allergen recognition by IgE triggers the degranulation of mast cells and basophils, releasing vasoactive mediators such as histamine triggering the allergic response. Therapeutic strategies such as oral immunotherapy (OIT) are effective at inducing desensitization in several allergens tested in FA through several mechanisms (5–14). However, these strategies require long-term continued consumption/exposure to the allergen to maintain desensitization (15). Preventative strategies such as early food introduction (16–20) and aggressive management of dry skin and eczema [a notable risk factor for the development of FA (21–23)] through emollient use (24–28) have potential for the prevention of FA development. However, these interventions require a more in-depth understanding of FA development to identify populations that would benefit from their use.

The development of FA is often linked to other atopic diseases such as AD which is common in infants and can lead to the development of other atopic diseases such as FA in a process called the atopic march (29). However, there is wide variability in the trajectories, clinical progression of FA, of infants through the atopic march, suggesting the presence of many different unidentified disease endotypes, molecular mechanisms driving the differences in disease trajectories, which are likely the result of environmental, genetic, epigenetic, and psychosocial factors (30, 31). For instance, classical FA has been classified as having 5 different disease trajectories (30). Persistent FA persists over time, whereas transient FA is outgrown as the patient gets older. In food-dependent exercise-induced allergy (FDEIA) exercise within 2–4 h after allergen consumption induces urticaria or anaphylaxis (30). In non-steroidal anti-inflammatory drug (NSAID) or aspirin-dependent and alcohol-dependent FA the intake of NSAIDs or alcohol prior to the ingestion of the allergic food increases the likelihood and severity of allergic reactions. Although different trajectories have been associated with differences in family history and clinical tests such as skin prick testing and allergen-specific IgE, the molecular mechanisms responsible for these different trajectories have not yet been identified. These studies highlight that although it is clear that that there are different disease trajectories and endotypes in FA, markers defining these different pathways have not been identified. For a more in-depth discussion of what is currently known about allergic trajectories please see other excellent reviews (30, 31).

The characterization of disease endotypes for FA is a key unmet need in the field. While there are differences in markers such as allergen-specific immunoglobulin G (sIgG) between different food allergens, such as between peanut (which is more likely to be persistent) and milk (which is more likely to be transient) (32), we currently lack the ability to predict which peanut or milk allergic patients will outgrow their FA. Therapies such as OIT are effective at inducing desensitization, however the long-time frame and high risk of (mostly mild) adverse reactions makes OIT an unnecessary burden for the few patients who have transient FA. Furthermore, while peanut OIT was able to induce desensitization in 85% of patients only 13% of patients achieved over 1 year of desensitization that does not require continued low-dose consumption of their allergenic food (sustained unresponsiveness, SU) (15). While it is thought that differences in molecular markers underlie these differences, currently we are unable to accurately predict which patients achieve either SU or desensitization. Therefore, there is a pressing need for a deeper characterization of the molecular changes that occur during the development of FA and in response to therapy. Omics technology has already proven to be a valuable tool for characterizing markers in FA (Table 1), however the application of these technologies to characterize FA development is still in its infancy. Here we summarize the application of omics technology in FA and discuss its future use in the characterization of FA development. For a broader understanding of omics assays in other contexts please refer to these excellent reviews (33–36).

## Genomics in FA development

Genomics is the study of changes in the DNA sequences as well as epigenetic changes that alter the accessibility or activity of the DNA, and is particularly useful for studying the heritable aspects and environmental influences (which often result in epigenetic changes) of FA. Family history is a known risk factor for the development of FA. The prevalence of self-reported peanut allergy in individuals with peanut allergic siblings is 6.9% (37), in contrast to ∼2.9% in the general population (38). Furthermore the peanut allergy concordance between monozygotic twins is 64.3% (39). These and other findings (2, 40–44) suggest that genetic and/or epigenetic modifications play a role in the development of at least some FA endotypes. Several studies have found associations between HLA genes and specific allergens, for instance in the LEAP trial the participants who consumed as opposed to avoided peanut had sIgG4 levels that were positively correlated with HLA-DQA1*01:02 (*P* = 2.21 × 10^–4^), suggesting a possible role in tolerance (45). However, the relationship between HLA-DQA1*01:02 may be context dependent as other studies have found that HLA-DQA1*01:02 is positively correlated with peanut allergy (OR 1.81) (46). In contrast, other HLA loci such as HLA-DR rs7192 and HLA-DQ rs9275596 are associated with peanut allergy (*P* = 5.5 × 10^−8^ and *P* = 6.8 × 10^−10^ respectively) (46, 47). Another HLA gene, HLA-DPB1∗02:01:02 as well as the single nucleotide polymorphism (SNP) rs9277630, were found to increase the risk of having wheat-dependent exercise-induced anaphylaxis in a Japanese patient cohort [odds ratio (OR): 4.13 and 3.53 respectively] (48). Aside from HLA genes, MALT1 and its single nucleotide variant rs57265082 was associated with the development of peanut allergy in patients who avoided peanut in the LEAP study (OR: 10.99) (49). Additionally, 2 STAT6 SNPs (rs324015 and rs1059513) were associated with the presence of FA, peanut allergy, and cow's milk allergy in a subgroup of the GENEVA cohort (50).

Alterations in epigenetic modifications such as DNA methylation have often been associated with the development of many diseases. DNA methylation profiling identified a panel of 16 CpG sites whose methylation pattern at birth is associated with IgE levels during childhood that were discovered in the Taiwan Maternal and Infant Cohort Study and validated in the Isle of Wight cohort (51), however whether this methylation panel is strong enough to predict the development of allergy has not been tested. In older patients, next generation bi-sulfite sequencing identified a panel of 12 CpG sites whose methylation status could be used to differentiate between peanut allergic and non-allergic patients (52). Most of these sites were associated with cytokines and other secreted proteins whose secretion was increased in peanut allergic patients. Similarly, a panel of 25 differentially methylated regions were found in infant blood from the Assessment of Lifestyle and Allergic Disease During Infancy (ALADDIN) cohort and replicated in the Children, Allergy, Milieu, Stockholm, Epidemiology (BAMSE) cohort that were associated with sensitization to food allergens at 5 years of age (53). In addition to sensitization, a panel of 203 CpG sites, with key hubs at NFKBIA and ARG1, has been identified whose methylations status is associated with differences in peanut reaction severity (54). Interestingly, methylation of these sites was suggested to be mediated by expression of PHACTR1 and ZNF121, suggesting that these genes could potentially be targeted to reduce reaction severity for at least peanut allergy.

In summary, most genomic assays in FA have compared polymorphisms or epigenetic modifications between allergic vs. non-allergic patients and have largely focused on the identification of risk factors for FA. Since genetic/epigenetic components likely influence FA development (2, 40–44), genomics could be used to compare differences at birth to the development of FA later in life. In comparison to other omics assays, genomics is especially valuable for identifying molecular mechanisms for FA population disparities and heritability. A large-scale international collaboration between birth cohort studies would be invaluable for validating whether individuals with different ethnic backgrounds are prone to following different trajectories, with differing underlying endotypes. International collaborations would not only allow for much larger cohort sizes but would also vastly improve the study's ability to sample differences in regional genetic variation which likely influence FA development. Twin studies are valuable for identifying factors associated with inheritance of FA, however the rarity of twins makes them difficult attain a large cohort size and would thus also benefit from large scale collaborations between institutions. This would facilitate the identification of high-risk patients that would benefit from preventative therapy.

## Transcriptomics in FA development

Transcriptomics is the study of RNA transcripts that bridge the gap between the genome and the proteome, which is useful for identifying active processes and pathways in the cell. These approaches tend to be sensitive, cheap, and high-throughput compared to other omics approaches which make them well suited for large-scale screening. Many studies have assessed alterations in the transcriptome response between patients with and without FA as well as in response to FA therapy (55–62). Recent studies have used whole blood transcriptomics to identify potential differentially regulated genes (DEGs) that are altered in peanut and tree nut allergies, however further studies are needed to characterize the predictive potential of these DEGs in an independent cohort (57, 60). RNA microarrays from peripheral blood mononuclear cells isolated from the blood of egg allergic patients found that after 3 months of OIT DEGs (linear fold change ≥|1.5| or ≥|1.3| with Benjamini–Hochberg adjusted *p*-value ≤ 0.05) were largely upregulated (72%) in comparison to baseline while after 8 months, most DEGs were downregulated (86%) (58). This could reflect changes in the immune response to OIT between short and long term OIT, however further studies are needed to functionally characterize these changes. Among transcriptomics assays, single cell RNA sequencing is particularly powerful for its ability to characterize the heterogenous population of cells and differentiate the complex response to therapy. This technology was well utilized in a study employing single cell RNA-seq and paired T cell receptor *α*/*β* (TCR*α*/*β*) sequencing to track CD154+ and CD137 + peanut-reactive T helper (Th) cells during OIT (61). Positive OIT outcome was associated with stronger suppression of Th2 signatures in Th2A-like cells, and poor OIT outcome was associated with a baseline inflammatory gene signature in Th1 and Th17 cells that did not change with OIT, suggesting that transcriptomics may be useful for the prognosis of FA. OIT was found to suppress the Th2 and Th1 gene signatures in effector T clonotypes, but not T follicular helper–like clonotypes. Single cell RNA-seq experiments from other cells (Tregs, CD4+ T cells, and *γδ* T cells) showed alterations in gene expression after peanut OIT (55, 56, 62). RNA seq during peanut OIT with adjuvant omalizumab in the FASTX study, found that the pretreatment of the anti-IgE antibody omalizumab prior to initiation of OIT does not alter whole blood gene expression in peanut allergic patients (59). However, a comparison of the whole blood gene expression profile before and after 2–3 years of omalizumab-facilitated peanut OIT showed that peanut OIT decreased processes associated with neutrophil degranulation. Although these studies suggest that there are differences in the gene expression profiles of patients with different desensitization outcomes, further studies are needed to identify a prognostic marker signature that could be used to predict these outcomes early on.

Analysis of the transcribed B cell receptor (BCR) sequences encoding allergen specific IgE in FA has recently contributed to understanding of FA pathogenesis. Since FA is usually driven by IgE-mediated reactions, characterization of IgE-producing could provide key insights into FA development. However, this has been challenging due to the rarity of B cells in FA patient blood, with B cell binding to sIgE epitopes being reported at 0.0097% (Ara h 1) and 0.029% (Ara h 2) (63–65). Allergen-specific IgE + B cells in peanut allergy are often members of clones containing other B cells expressing IgG or IgA subclass isotypes, and current evidence suggests that the IgE + cells are a minority of total allergen-specific B cells, even in symptomatic FA patients(63, 66). Single-cell transcriptomic analysis of IgE + B cells in peanut allergic patients identified a peanut allergen-specific public or convergent clonotype with highly similar BCR sequences between two allergic individuals, as well as additional clones with similarity to BCR heavy chain transcripts from an unrelated study of peanut allergic patients undergoing OIT, indicating that some portion of the peanut allergen-specific IgE response is shared between individuals at the level of BCR sequences (64, 67). Beyond the blood, initial studies of IgE + B lineage cells in the esophagus, stomach and duodenum of peanut allergic patients showed evidence of local clonal expansion and IgE class switching in these tissues, as well as additional convergent or public IgE + clonotypes shared between patients (63). The extent to which mast cells in these tissue sites acquire locally produced IgE in their high-affinity IgE receptors, and the functional consequences for mast cell sensitization and degranulation warrant further investigation. Other intriguing and largely open questions in FA research are how OIT and other immunotherapies alter the clonal populations of allergen-specific B cells in terms of clonal frequencies, phenotypes, isotype expression and affinity maturation.

Although transcriptomics has been used to characterize alterations in immune cell populations after therapy, the use of transcriptomics to identify early prognostic markers for the development of FA is still in its infancy. The high throughput, sensitivity, comparatively low cost, and potential for single cell analysis makes transcriptomics particularly amenable for the identification of novel prognostic markers. These traits make transcriptomics particularly valuable for the identification of markers from specific cell populations that are particularly important for FA. Analysis of antigen-specific B cell and T cell receptor transcripts in FA is beginning to make inroads towards greater understanding of the features of B cell and T cells populations that ultimately result in production of pathogenic IgE, as well as providing a high-resolution approach toward monitoring the effects of OIT and other therapies on adaptive immune cell populations in FA patients. Other exciting cell populations for elucidating FA endotypes that are currently understudied in FA include basophils and mast cells, both of which are being investigated for their potential in predicting therapeutic outcomes. However, general transcriptome assays have a high false discovery rate, and future studies should ensure that transcriptomics leads are validated at least at the RNA level, and ideally at the protein level, by assays such as qPCR and Western blot.

## Proteomics in FA development

Not all RNA transcripts are translated into functional proteins therefore, out of all the single-target omics approaches, proteomics provides the strongest predictive power (68), likely due to proteins being one of the closest measures for biological activity in the cell. Although many studies have used proteomics for the identification of novel allergenic peptides (69–74), only a few studies have leveraged the power of proteomics for FA development.

Lasso logistic regression modeling of whole blood proteomics from a small sub group of the FARMFLORA cohort identified a panel of 27 proteins and in blood collected at 1 month of age whose abundance was associated with the development of FA by 8 years of age(AUC 0.87) (75). Since AD often precedes the development of FA, alterations in the skin may reflect the development of FA later in life. Skin tape strip proteomics indicated that patients with both AD and FA have increased abundance of several keratins, notably KRT5, KRT14, and KRT16 in comparison to healthy or patients with only AD (76). This increase in KRT5, KRT14, and KRT16, was found to be a stronger predictor for the co-occurrence of AD and FA than other parameters including transepidermal water loss, and factors identified from either transcriptomics or metabolomic, highlighting the strength of proteomics for the identification of prognostic markers. In a follow up study, a proteomic analysis of skin tape strips in children identified a panel of 45 proteins, including the previously identified keratins, that were associated with high transepidermal water loss and allergic sensitization (77). This panel was validated in a separate cohort of adult patients, where the protein panel was significantly higher in AD patients with peanut allergy and was lowest in healthy patients.

Together these studies show that proteomics can identify markers for FA trajectories, in this case for patients who develop AD and FA. Although this protein panel would likely need to be further refined for better separation between patients with both AD and FA and patients with only AD, before it is used in the clinic, these studies highlight the potential of applying proteomics to identify markers for other indications such as FA development. Of particular interest is the potential of skin tape strip proteomics, as they are minimally invasive and can easily be performed on infants. This would allow them to be easily implemented in birth cohort studies that follow the development of FA. However further studies on the use of proteomics on FA alone, outside the context of AD, are needed to assess whether they retain a similar predictive potential. Another interesting application of this technology is in studies investigating the treatment of AD for the prevention of FA. These studies have been hampered by adherence issues with the use of emollients for treating AD. Skin tape stripping could be used to compare skin proteomic signatures to levels of the emollient that were used on the skin, as has been done for other topical drugs like tazarotene (78), by analyzing skin tape strips from different layers. This could help link actual use of emollients for the treatment of AD to molecular alterations in skin cells that could influence FA development.

## Metabolomics/lipidomics in FA development

Metabolomics and its subfield focused on lipids, lipidomics, studies the metabolic products formed when active proteins are broken down which are often altered in disease states. Metabolomics from biofluids has provided key insights into the underlying mechanisms in many diseases, and recently several studies have identified potential FA metabolic prognostic markers from a range of biofluids including plasma, cord blood, saliva, and urine. The Barwon Infant Study found that higher plasma phenylalanine was associated with increased odds of developing FA (OR 1.6, 95% CI: 1.092–2.344) in 1 year old (yo) infants (79). Furthermore, patients with peanut allergies have elevated lactate, creatinine, and glutamine which could be detected in their plasma prior to consumption of peanuts, suggesting that alteration in metabolite levels may be able to predict the presence of FA (81). In line with this, untargeted metabolomics identified a panel of 53 metabolites that are altered in children with FA compared to non-atopic children, including lipid metabolites such as sphingolipids and ceramides and amino acid metabolites such as lysine and threonine (82). Interestingly, 41 metabolites were significantly altered in children with multiple, as opposed to single FA, and some metabolites were altered in children who had anaphylactic reactions. Similarly, serum metabolomics identified 12 metabolites that were differentially abundant in FA and control children including sphingolipids, acylcarnitine, and lysophosphatidic acid metabolites, which were used to build a random forest model for the prediction of FA (AUC 0.708, 95% CI 0.483–0.926) (83). Interestingly, 12 metabolites were differentially abundant between children with persistent vs. transient FA, which could be used for the prediction of FA resolution (AUC 0.947, 95% CI 0.748–1.000). However further studies are needed to validate this model in a large independent cohort.

In addition to serum/plasma other studies are starting to investigate the metabolome of other biofluids. The FARMFLORA birth cohort found 8 cord blood metabolites that were associated with the eventual development of FA, however no single metabolite was associated with FA development across all time points (18 months, 3 years, and 8 years) possibly due to the low sample size in this cohort (*n* = 44) (84). In contrast, lipidomics from cord blood samples in the Boston Birth Cohort identified several triacylglycerols that were decreased in children who developed FA with C56:8 having the strongest association (OR 0.57, 95% CI: 0.42–0.77) (85). A study of adult twins with FA found 97 metabolites that were differentially altered in fecal samples of healthy and allergic twins (86). Interestingly, the strongest sub-pathway in these metabolites was diacylglycerol, which was enriched in healthy as opposed to FA twins. Urinary metabolomics from a milk allergic mouse model found that tetranor-prostaglandin D metabolite (tetranor-PGDM) was in increased in FA mice and was associated with disease severity (87). These findings were validated in humans where urinary tetranor-PGDM was significantly increased in human patients with FA in comparison to healthy controls (*P* < 0.001) and other atopic diseases. Liquid chromatography/mass spectrometry from saliva samples found that peanut allergic patients had decreased acetate, butyrate and propionate in comparison to healthy controls (88), highlighting that saliva may be an easy to collect source of FA biomarkers.

Together these findings demonstrate that metabolomics holds great potential for the characterization of FA development, with several studies identifying potential markers for predicting FA development and resolution (79, 82, 84, 85). Importantly, these studies highlight the importance of building a panel of prognostic biomarkers, as the predictive power of individual markers was generally not very powerful. Future studies investigating the durability of these prognostic markers in larger cohorts across more diverse age groups would facilitate the translation of these exciting findings to the clinic (Table 1). Other key questions of interest are whether these markers are better at predicting the trajectories of one allergen vs. another and whether metabolic differences could be used to predict response to therapy. For instance, the metabolism of patients with peanut allergy may be notably different than those with milk allergy given the differences in their resolution. These questions would require a very large patient cohort, highlighting the importance of large-scale international collaborations.

**Table 1:** Markers from omics studies for FA and FA development.

Marker	Omics Branch	Population	Association	Citation
HLA-DQA1*01:02	Genomics	LEAP study, 640 4–11 mo with severe eczema and/or egg allergy	Positively correlated with peanut sIgG4 levels (*P* = 2.21 × 10–4)	(45)
HLA-DQA1*01:02	Genomics	73 peanut allergic and 148 non-allergic ∼1 yo infants	Positively correlated with presence of peanut allergy (OR 1.81)	(46)
HLA-DR rs7192 and HLA-DQ rs9275596	Genomics	Chicago Food Allergy Study, 1,315 children; 1,444 biological parents	Associated with the presence of FA (*P* = 5.5 × 10^−8^ and *P* = 6.8 × 10^−10^)	(47)
HLA-DPB1∗02:01:02 and rs9277630 (HCG24 SNP)	Genomics	77 individuals with WDEIA	Increases the risk of having wheat-dependent exercise-induced anaphylaxis (OR: 4.13 and 3.53 respectively)	(48)
MALT1 and rs57265082 (MALT1 SNP)	Genomics	LEAP study, 640 4–11 mo with severe eczema and/or egg allergy	Associated with the development of peanut allergy in patients who avoided peanut (OR: 10.99)	(49)
rs324015 and rs1059513 (STAT6 SNPs)	Genomics	Subgroup of the GENEVA cohort, 369 trios of children with FA and their patents, including 262 children	Associated with the presence of FA (*P* = .036 and *P* = .013)	(50)
Methylation of 16 CpG site panel	Genomics	Taiwan Maternal and Infant Cohort Study, 299 mother-newborn	Methylation pattern at birth is associated with IgE levels during childhood	(51)
Methylation of 12 CpG sites	Genomics	10 peanut allergic and 10 non-allergic 5–10 yo	Methylation status differentiates between peanut allergic and non-allergic patients	(52)
Differential methylation of 25 regions	Genomics	ALADDIN cohort, 288 PBMC samples from 74 mother/child pairs	Associated with sensitization to food allergens at 5 years of age	(53)
Methylation of panel of 203 CpG sites	Genomics	21 peanut-allergic children	Associated with differences in peanut reaction severity	(54)
CD154+ and CD137 + peanut-reactive Th cells during OIT	Transcriptomics	12 peanut allergic patients	Positive OIT outcome associated with suppression of Th2 signatures and poor OIT outcome associated with a baseline inflammatory gene signature in Th1 and Th17 cells that did not change with OIT	(61)
Lasso regression modeling of a protein panel: HLA A-C/E-G, IL-9, GADD45A, MPO, IL-15, TNFAIP3, MYDGF, MMP9, CCR10, IL-17A, EPX, CXCL9, IRF2, CCL8, IL-26, CCL17, PTGDR, LGALS 9/9B/9C, HRG, IL-12B, EHBP1, farming environment, CCL24, NGF, PLEKHA4, VEGFD, CXCL5, and IGIP	Proteomics	Subgroup of 8 children from the FARMFLORA birth cohort	Abundance at 1 month of age predicts development FA by 8 years of age (AUC 0.87)	(75)
Skin KRT5, KRT14, and KRT16	Proteomics	AD FA+, *n* = 21; AD FA−, *n* = 19; NA, *n* = 22	Increased in patients with both FA and AD	(76)
Panel of 45 proteins	Proteomics	AD FA+, *n* = 21; AD FA−, *n* = 19; NA, *n* = 22	Associated with high transepidermal water loss and allergic sensitization	(77)
Plasma phenylalanine	Metabolomics/Lipidomics	Subcohort of 485 infants from Barwon Infant Study birth cohort	Associated with increased odds of developing FA (OR 1.6, 95% CI: 1.092–2.344)	(79)
Increase in several unsaturated fatty acids and decrease in conjugated bile acids	Metabolomics/Lipidomics	3–36 mo children, 42 AD and 23 healthy controls	Associated with FA	(80)
Lactate, creatinine, and glutamine	Metabolomics/Lipidomics	23 participants (12 peanut allergic and 11 peanut tolerant)	Elevated in patients with peanut allergy	(81)
Panel of 53 metabolites	Metabolomics/Lipidomics	125 ≤ 12 yo children	Altered in children with FA	(82)
Random forest model built from 12 metabolites	Metabolomics/Lipidomics	20 FA and 20 healthy children	Prediction of FA (AUC 0.708, 95% CI 0.483–0.926) and FA resolution (AUC 0.947, 95% CI 0.748–1.000)	(83)
Cord blood metabolite panel: opthalmic acid, ursodeoxycholic acid, *δ*-tocopherol, glyceric acid, lactose, cellobiose, sorbitol, and nigerose	Metabolomics/Lipidomics	FARMFLORA birth cohort, 65 children	Associated with the development of FA at any timepoint (18 months, 3 years, and 8 years)	(84)
Triacylglycerols	Metabolomics/Lipidomics	Boston Birth Cohort, 647 mother-child pairs	Decreased in children who developed FA with C56:8 having the strongest association (OR 0.57, 95% CI: 0.42–0.77)	(85)
Panel of 97 fecal metabolites	Metabolomics/Lipidomics	18 adult twins with discordant (13) or concordant (5) FA	Differentially altered in FA vs healthy twins	(86)
Urinary tetranor-PGDM	Metabolomics/Lipidomics	9 FA patients and 39 healthy control	Increased in FA	(87)
Salivary acetate, butyrate and propionate	Metabolomics/Lipidomics	105 subjects (56 with peanut allergy and 49 healthy subjects)	Decreased in peanut allergy	(88)

Large scale omics approaches have provided an invaluable resource for discovering the disease development and druggable targets in many different diseases (89–91), and technological advances continue to make these technologies more powerful and affordable. To date, the use of genomics, transcriptomics, proteomics, metabolomics, and lipidomics have been more focused on identifying biomarkers for the presence or absence of FA, however application of these technologies could also be used for the characterization of FA development. This would not only aid in the identification of patients that are likely to have persistent FA who are more likely to benefit from therapeutic options but could also help predict which patients are at risk for developing other atopic diseases.

## Using multi-omics approaches to characterize FA development

**Figure 1:**
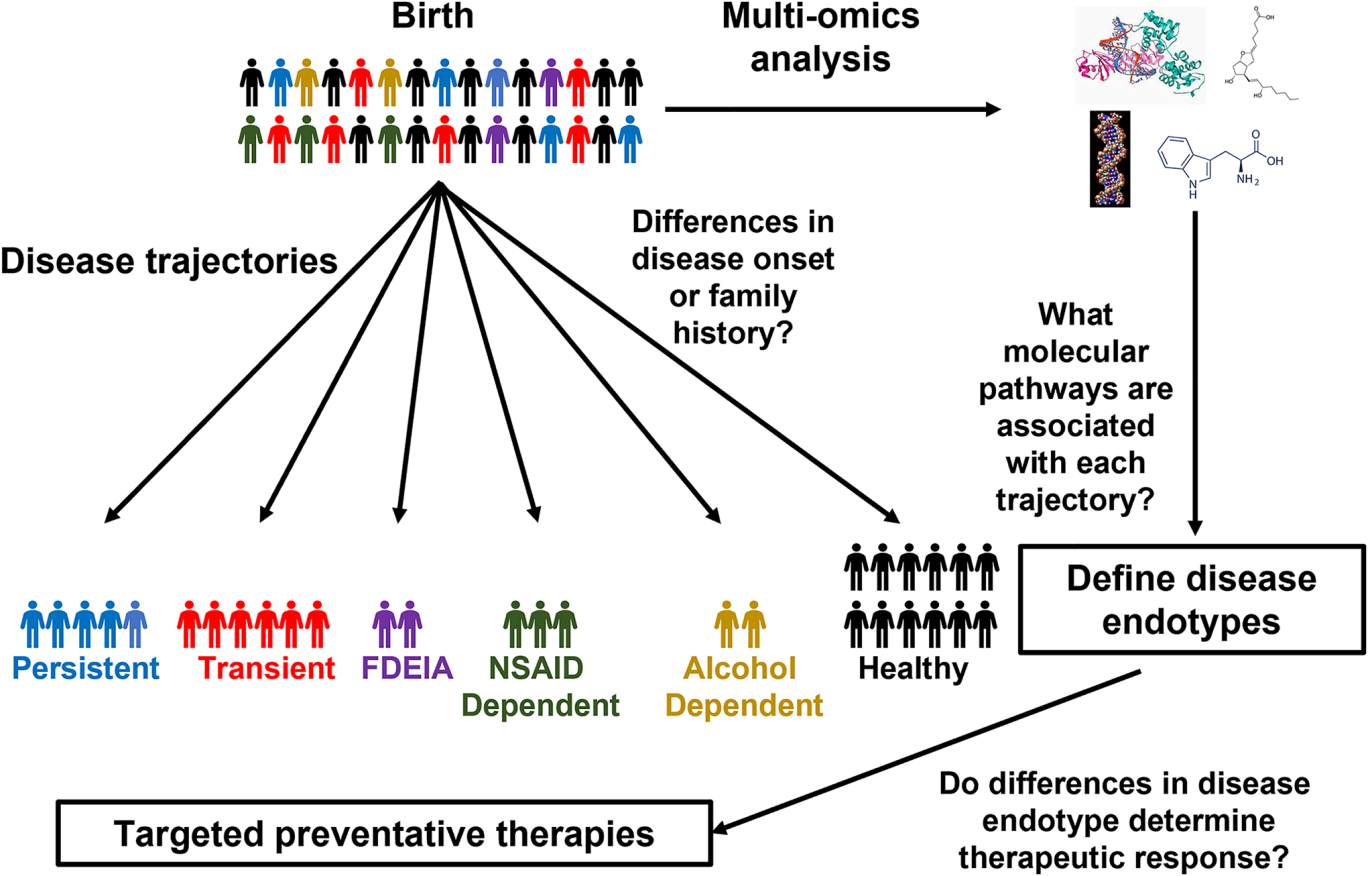
Using birth cohorts to unravel FA endotypes. Schematic of how omics approaches could be applied to birth cohorts to characterize the molecular mechanisms of FA development.

So, when should these omics technologies be applied to FA to assess FA development? The landmark Learning Early About Peanut (LEAP) trial as well as follow-up studies demonstrated that early introduction of peanut can prevent the development of peanut allergy even in sensitized children (19, 92, 93), indicating that the risk of FA begins developing early in life. Therefore, dissecting the mechanisms governing the heterogeneity FA development, would require longitudinal monitoring of patients from close to birth until later in life, making large scale birth cohort studies most suitable for identifying markers for different FA trajectories and endotypes (Figure 1). Due to the challenges in recruiting a large diverse patient cohort at a single center, large international collaborations between birth cohort studies could greatly enhance our ability to characterize FA development. Although combining multiple cohorts does increase the complexity of the data, tools have been developed to facilitate the integration of patient cohorts (94, 95). Once it is clear what trajectories patients follow, samples collected early in life could be profiled to identify predictive markers for the stratification of patients based on disease trajectories and identification of individuals who would benefit from preventative therapies (24, 96) (NCT03742414, NCT04798079). Although most studies, in FA to date have focused on single omics approaches a combined multi-omics approach using machine learning allows for the development of a much stronger predictive model than any single approach alone (68). This highlights the importance of collaboration across groups with different research backgrounds as different omics approaches each have their own unique advantages in characterizing FA development. Future studies should work closely with international consortia to establish standardized methodologies for FA diagnosis, sample collection, and treatment. This would greatly facilitate large-scale collaborations which are often challenging due to the lack of standardized practices. Furthermore, the generation of large-scale publicly available sample biobanking would provide a tremendous resource for dissecting molecular mechanisms of FA development as well as many other crucial research questions.

## Conclusion

In the last several years there has been an increasing application of omics technology in FA demonstrating a strong rational to leverage these powerful technologies for the identification of FA trajectories and endotypes. Although there have been relatively few studies so far, they have already uncovered many exciting leads that with further development could allow for the prediction of which patients are likely to develop FA and would be greatly benefit from preventative strategies. The potential markers for FA resolution could be further developed to identify which patients are most likely to benefit from therapies such as OIT. Lastly, preliminary markers for therapeutic outcomes could lead to the development of next generation therapies to improve the ability to achieve sustained desensitization. We look forward to the future application of these technologies in large-scale longitudinal birth cohorts and would like to encourage the use of large-scale international collaborations to allow for rigorous assessment of these complex challenges.
