# Corrigendum: Advances and potential of omics studies for understanding the development of food allergy

**DOI:** 10.3389/falgy.2024.1373485

**Published:** 2024-02-23

**Authors:** Sayantani B. Sindher, Andrew R. Chin, Nima Aghaeepour, Lawrence Prince, Holden Maecker, Gary M. Shaw, David Stevenson, Kari C. Nadeau, Michael Snyder, Purvesh Khatri, Scott D. Boyd, Virginia D. Winn, Martin S. Angst, R. Sharon Chinthrajah

**Affiliations:** ^1^ Sean N. Parker Center for Allergy and Asthma Research at Stanford University, Palo Alto, CA, United States; ^2^ Department of Anesthesiology, Perioperative and Pain Medicine, Stanford University School of Medicine, Palo Alto, CA, United States; ^3^ Department of Pediatrics, Stanford University School of Medicine, Palo Alto, CA, United States; ^4^ Department of Biomedical Data Science, Stanford University School of Medicine, Palo Alto, CA, United States; ^5^ Department of Medicine, Institute for Immunity, Transplantation and Infection, Stanford University School of Medicine, Palo Alto, CA, United States; ^6^ Department of Genetics, Stanford University, Palo Alto, CA, United States; ^7^ Department of Pathology, Stanford University, Palo Alto, CA, United States; ^8^ Department of Obstetrics and Gynecology, Stanford University School of Medicine, Palo Alto, CA, United States

**Keywords:** food allergy (FA), genomics, proteomics, transcriptomics, metabolomics, disease development

A Corrigendum on Advances and potential of omics studies for understanding the development of food allergy Sindher SB, Chin AR, Aghaeepour N, Prince L, Maecker H, Shaw GM, Stevenson DK, Nadeau KC, Snyder M, Khatri P, Boyd SD, Winn VD, Angst MS and Chinthrajah RS (2023) Advances and potential of omics studies for understanding the development of food allergy. Front. Allergy 4:1149008. doi: 10.3389/falgy.2023.1149008

**Text Correction**

In the published article, there was an error in the section **Metabolomics/lipidomics in FA development**. The sentence “Untargeted serum metabolomics from infants identified an increase in several unsaturated fatty acids, such as free fatty acid (FFA) 16:1 (palmitoleic acid) and FFA 20:1 (eicosenoic acid), and a decrease in conjugated bile acids, such as glycocholic acid and taurocholic acid, in infants with FA (80).” [80: Huang et al., 2014] was referring to a study in atopic dermatitis as opposed to food allergy. This sentence has been removed from the manuscript.

The authors apologize for this error and state that this does not change the scientific conclusions of the article in any way. The original article has been updated.

